# Hospitalized Patients’ Sleep Quality Compared Between Multioccupancy Rooms and Single-Patient Rooms

**DOI:** 10.1177/19375867231168895

**Published:** 2023-05-04

**Authors:** Laura Schafthuizen, Erwin Ista, Marianne van der Heijden, Liesbeth van Heel, Jill Maben, Joost van Rosmalen, Casper H. J. van Eijck, Monique van Dijk

**Affiliations:** 1 Department of Internal Medicine, section Nursing Science, Erasmus MC, Erasmus University Medical Center, Rotterdam, The Netherlands; 2 Erasmus MC, Erasmus University Medical Center, Rotterdam, the Netherlands; 3 School of Health Sciences, University of Surrey, Guildford, UK; 4 Department of Biostatistics, Erasmus MC, Erasmus University Medical Center, Rotterdam, the Netherlands; 5 Department of Epidemiology, Erasmus MC, Erasmus University Medical Center, Rotterdam, the Netherlands; 6 Department of Surgery, Erasmus MC, Erasmus University Medical Center, Rotterdam, the Netherlands

**Keywords:** hospitalized patients, sleep quality, healing environment, actigraphy, Richards–Campbell sleep questionnaire

## Abstract

**Objectives:**

To evaluate patients’ sleep quality in a former hospital with two-and four-bedded rooms compared to a new hospital that incorporated evidence-based design features, including exclusively single-patient rooms (SPRs).

**Background:**

Hospitalized patients often report poor sleep quality due to both patient-related factors and hospital environmental factors. It is unclear if staying in an SPR in a hospital designed as a healing environment is associated with better sleep quality.

**Methods:**

In a before-after study, sleep quality, duration, and efficiency over 72 hr were measured with a sleep diary, GENEActiv accelerometer, and the Richards–Campbell Sleep Questionnaire (RCSQ) with scores ranging from 0 to 100, with higher scores reflecting better sleep. Participants were either staying alone in the former hospital with two-and four-bedded rooms (Group 1), sharing a room with one to three fellow patients (Group 2), or staying alone in a newly designed hospital with 100% SPRs (Group 3).

**Results:**

We included 17 patients in Group 1, 32 patients in Group 2, and 56 patients in Group 3. Univariable linear mixed model analysis, controlling for night number, revealed that the RCSQ total score was lowest in Group 2 compared to the other two groups. In the multivariable analysis, the RCSQ score was also the lowest in Group 2, with a significant effect from covariate “use of night medication.”

**Conclusion:**

Self-reported sleep quality of hospitalized patients in a hospital with 100% SPRs designed as a healing environment was slightly better than that of patients staying in multioccupancy rooms with fellow patients.

Healthy sleep is essential for any individual to perform normal activities and is a fundamental health-related factor for all ages (Park & Kim, 2017). Healthy sleep requires adequate duration, good quality, appropriate timing, and regularity (Watson et al., 2015). The opposite, poor sleep, may lead to increased blood pressure, hormonal irregularities, increased glucose metabolism, and inflammations (Abrams, 2015). Also, sleep deprivation has been associated with depression and negative mood (Van Dongen & Dinges, 2005).

Measuring sleep quality is difficult due to its imprecise definition (Krystal & Edinger, 2008). Kline (2013) defined sleep quality as “one’s satisfaction of the sleep experience, integrating aspects of sleep initiation, sleep maintenance, sleep quantity, and refreshment upon awakening.” In research, both objective and subjective methods are applied for measuring sleep quality, with polysomnography as the current golden standard in objective measurement (Krystal & Edinger, 2008). However, polysomnography is impractical, invasive, and expensive (Kaplan et al., 2017). Thus, researchers have turned to alternative, cost-effective ways of evaluating sleep quality, such as sleep diaries and wrist actigraphy (Moore et al., 2015). Sleep experts recommend both sleep diaries and actigraphy to assess different aspects of an individual’s sleep experience (Vitiello et al., 2004)

Hospitalized patients often self-report poor sleep quality (Kulpatcharapong et al., 2020; Lei et al., 2009; Missildine et al., 2010; Wesselius et al., 2018). The reasons are multifactorial and—apart from several patient-related factors, such as pain, anxiety, and clinical symptoms—include hospital environmental factors as well (Ding et al., 2017; DuBose & Hadi, 2016). Noise nuisance—often produced by other patients—has been found a contributing factor for poor sleep quality (Delaney et al., 2019; Garside et al., 2018; Wesselius et al., 2018). Other causes are excessive light exposure during the night as well as nightly medical and nursing interventions, such as measuring vital signs and overnight IV fluids (Gimenez et al., 2017; Morse & Bender, 2019; Oléni et al., 2004). Furthermore, organizational factors such as early awakenings and ward rounds during night-time could negatively affect sleep quality (Wesselius et al., 2018; Yilmaz et al., 2012).

Attempts to improve the quality of hospitalized patients’ sleep have mainly focused on pharmacological interventions, despite studies indicating that organizational and environmental modifications can also improve this (Maben et al., 2016; Villarosa et al., 2019). Creating a so-called “healing environment” might positively impact patients’ sleep quality (Ulrich et al., 2008). A healing environment emphasizes patient privacy, acoustic comfort—for example, doors closed and sound-absorbent ceiling—and patient control over bed position, room temperature, lighting, and sounds (DuBose & Hadi, 2016; Fietze et al., 2016). A cross-sectional study found that patients in single-patient rooms (SPRs) slept longer than patients in multioccupancy rooms (Dobing et al., 2016). In a survey study, almost 85% said to prefer staying in a single room—with privacy as the main reason (Reid et al., 2015). Similarly, in the mixed methods case study of Cusack et al. (2019), 45 of the 48 patients (93.8%) anticipated that sleep and rest would be better in SPRs. Infection prevention is another reason why SPRs in newly built hospitals nowadays are more and more the standard (Halaby et al., 2017).

There is a moderate level of evidence to support the assumption that a well-designed “healing” hospital environment can contribute to patients’ sleep quality, but data obtained through objective measures are lacking.

The aim of this study is to compare patients’ sleep quality in a newly designed hospital, with that of patients staying in a former hospital with multioccupancy rooms without healing environment concepts. We hypothesized that patients in SPRs report better sleep quality than patients staying in multioccupancy rooms.

## Method

### Design

An uncontrolled before-after study, embedded in a larger study (the WELCOME study) examining staff and patients’ perceptions of various aspects of the new hospital of Erasmus University Medical Center Rotterdam, the Netherlands. The Medical Ethics Review Board of Erasmus MC approved the study protocol (MEC-2017-1103).

### Setting and Participants

In May 2018, Erasmus University Medical Center was relocated to a newly built hospital adjacent to the old building. The former hospital originated from the early 1960s and offered a double corridor ward configuration with either two- or four-bedded rooms. A shared bathroom was located in the corridor, up to 20 m from the patient rooms. No special noise reducing features were present. The new hospital was designed according to a healing environment approach, with exclusively SPRs with an ensuite bathroom. In the new hospital, patients have control over the lighting, room temperature, and air quality. Noise-reduction features were taken by mounting acoustic ceiling tiles, laying rubber flooring, and installing solid, sound-reducing doors. A small, soft light shining on the floor on the side of the bed en route to the bathroom provides orientation at night. Furthermore, the room has a friendly decor with designer furniture and is painted in a calming color. To facilitate social contact for the patients, visiting hours are extended and a fold-out sofa bed provides the opportunity of rooming-in.

Adults patients able to provide informed consent, accommodated in either internal medicine or surgical wards with an expected stay of at least three nights, were eligible for inclusion. Excluded were patients from intensive care units and stroke units, as well as patients with a delirium, confusion, or reduced level of consciousness.

## Measurements

### Richards–Campbell Sleep Questionnaire (RCSQ)

The RCSQ is a self-report questionnaire that assesses five dimensions of sleep: sleep depth, sleep latency, awakenings, returning to sleep, and sleep quality (Richards, 1987; Supplemental Table 1). Each dimension is scored from 0 to 100. The mean of these five-dimension scores, known as the “total score,” represents the overall perception of sleep quality. A total score between 0 and 25 indicates *very poor sleep*, while 26–50 indicates *poor sleep*, 51–75 *good sleep*, and 76–100 *very good sleep* (Karaman Ozlu et al., 2018).

#### Sleep diary

Patients recorded in a diary the hospital-related, personal, and environmental factors that disturbed their sleep during the night. The following parameters were collected from the sleep diaries: time to bed, sleep latency, nocturnal awakenings, and waking up time. Sleep latency is defined as the time it takes to fall asleep after turning lights out. On average, healthy persons fall asleep after 10–20 min (Littner et al., 2005). Nocturnal awakening is defined as waking up from any stage of sleep. A European study in the general population found that most of the individuals (46.0%) awakened only once during the night (Ohayon et al., 2017). A complete overview of sleep parameters is presented in Supplemental Table 1. In addition, patients recorded whether or not they had been woken up by a nurse in the morning, and pain severity over the last 24 hr, graded on a numeric rating scale (NRS) of 0 (*no pain*) to 10 (*the worst pain possible*; Thong et al., 2018). Patients were also asked to record “night medication” defined as medication that could impact sleep. Included were benzodiazepines, haloperidol, and opioids.

### Accelerometry

Objectively measured sleep characteristics were collected from triaxial, wrist-worn GENEActiv accelerometers (Activinsights, Kimbolton, Cambs, UK; Supplemental Table 1). The GENEActiv accelerometer has been validated for measuring sleep duration and efficiency and has been used in previous studies to assess sleep in adults (te Lindert & Van Someren, 2013; van Hees et al., 2015). Van Hees et al. (2015) proposed to determine sleep duration from the sustained inactivity bouts, where the estimated angle of the accelerometer relative to gravity does not change beyond 5° for at least 5 min (van Hees et al., 2015). Inadequate sleep efficiency was defined as <85% and short sleep duration as <7/24 hr (Ohayon et al., 2017).

### Munich Chronotype Questionnaire

Chronotype is the natural inclination to sleep at a certain time. Besides regulating sleep and wake times, chronotype has an influence on appetite, exercise, and core body temperature (Fischer et al., 2017). Early chronotype persons (advanced sleep period; most active and alert in the morning) and late chronotype persons (delayed sleep period; most active and alert in the evening) are the two extremes, while normal chronotype persons have some flexibility in the timing of their sleep period (Lack et al., 2009). The 19-item Munich Chronotype Questionnaire examines wake and sleep patterns on both work and free days, energy levels throughout the day, sleep latency, and exposure to daylight (Roenneberg et al., 2003). On the basis of the data from the Munich Chronotype Questionnaire, a classification into early, normal, and late chronotypes is derived.

### EuroQol-5D-3L (EQ-5D-3L) and EQ-VAS

The Dutch versions of the EQ-5D-3L and the EQ-VAS were applied to assess the participating patients’ health state (Buchholz et al., 2018). The EQ-5D-3L includes five dimensions of health state: mobility, self-care, usual activities, pain/discomfort, and anxiety/depression. The items are assessed with a three-level scale (no/some/extreme problems). This results in a health state index score ranging from −0.329 (*worst possible health state*) to 1 (*perfect health*) according to the Dutch EQ-5D tariff (Versteegh et al., 2016). The EQ-VAS measures self-reported current health on a visual analogue scale from 0 (*worst imaginable health state*) to 100 (*best imaginable health state*). The EQ-5D-3L and EQ-VAS have shown to be valid, reliable, and responsive in many situations and populations (Buchholz et al., 2018).

## Data Collection

Data collection took place in two periods: from March 2018 to May 2018 (former hospital) and February 2019 to April 2019 (new hospital). The research coordinator (LS) trained six research assistants on the data collection procedures. They visited the eligible wards during daytime on weekdays and first asked the nurse in charge which patients were eligible. These patients were invited to participate in the study and provided written informed consent. In this way, a convenience sample of patients was developed. They completed the Munich Chronotype Questionnaire and the EQ-5D-3L. In addition, patients were asked to wear a GENEActiv accelerometer for three consecutive nights on their nondominant wrist. During this period, we also collected the RCSQs and sleep diaries each morning. The research assistants or, if possible, patients themselves entered the answers on a tablet. Data were retrieved with Microsoft® Excel 2016 and stored on a secure server.

## Statistical Analysis

Categorical data were presented as numbers and percentages. Normally distributed continuous variables were presented as mean and standard deviation; nonnormally distributed variables as median and interquartile range (IQR). Patient characteristics were compared between groups with Fisher exact tests for categorical variables. For continuous variables, the Kruskal–Wallis H tests were applied as appropriate. Considering that one third of participants in the former hospital had no fellow patients, we created three groups: 1 = *former hospital, without fellow patients*; 2 = *former hospital, with one to three fellow patients*; and 3 = *new hospital without fellow patients*.

We applied two linear mixed models; Model 1 with group (Groups 1–3) and night of assessment (Night 1–3) as predictor variables and Model 2 with the same predictor variables and additional covariates: chronotype (early, normal, and late), EQ5D-3L index score, use of sleep medication, length of stay (from admission until first day of assessment), and self-reported NRS pain. A random intercept was used to account for the within-patient correlations. Outcome variables for Model 1 were the RCSQ five subscale scores, and outcomes variables for both Models 1 and 2 were the RCSQ total score and sleep efficiency derived from accelerometry.

The health status index scores EQ5D were calculated with R-package “EQ5D” (https://cran.r-project.org/web/packages/eq5d/index.html). Sleep characteristics were derived from raw accelerometer data and processed using the open-source R-package “GGIR,” Version 2.3-01 (https://cran.r-project.org/web/packages/GGIR/index.html), which has been validated against data from polysomnography (van Hees et al., 2015). All other data were analyzed using IBM SPSS Statistics for Windows Version 25.0 (IBM Corp., Armonk, NY).

## Results

**Figure 1.**
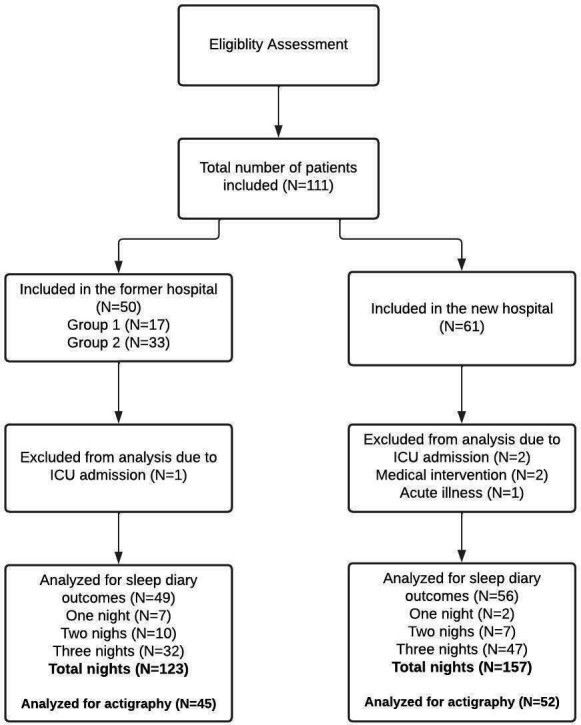
Flowchart.

We included 111 patients in total, 50 in the former hospital (Group 1, *N* = 17, Group 2, *N* = 33), and 61 in the new hospital (Group 3). Due to transfer to ICU, acute deterioration, or medical interventions, data of six patients were excluded from analysis; one in the former hospital and five in the new hospital (Figure 1). In total, we collected data over 123 nights in the former hospital in 49 patients and over 157 nights in 56 patients in the new hospital. Data collection was not completed for all measurements. Accelerometer data were collected in 45 patients in the former hospital and in 52 patients in the new hospital. Furthermore, not all sleep diaries were completed for all three nights. The flowchart in Figure 1 gives an overview of the included patients and number of nights that were analyzed.

### Demographic and clinical characteristics of patients

The proportion of surgical patients differed significantly between groups with three (17.6%) surgical patients in Group 1, nine (28.1%) in Group 2, and 29 (51.8%) in Group 3 (*p* = .013) Median age did not significantly differ between groups. Use of night medication did not significantly differ between patients in the former hospital (Groups 1 and 2) compared to the new hospital (Group 3; *p* = .890). Four patients in the former hospital and two patients in the new hospital took opioids or antipsychotics as well as medication to promote sleep. Most patients were early chronotypes (respectively, 82.4%, 86.2%, and 87.5%). The EQ5D-3L index score differed significantly between groups, with median values of 0.75 in Group 1, 0.81 in Group 2, and 0.67 in the new hospital (*p* = .030)—mainly due to differences on subscale “usual activities” (Table 1).

**Table 1.** Demographic and Clinical Characteristics of Included Patients.

Demographic and Clinical Characteristics	Group 1 (*n* = 17)	Group 2 (*n* = 32)	Group 3 (*n* = 56)	*p* Value
Age in years, median (IQR)	64 (54–70)	58 (38–67)	61 (47–69)	.477
Male, *N* (%)	10 (58.8)	24 (75.0)	32 (57.1)	.235
Type of patients, *N* (%)				.013
Surgical	3 (17.6)	9 (28.1)	29 (51.8)	
Internal medicine	14 (82.4)	23 (71.9)	27 (48.2)	
Specialties, *N* (%)				.005
Gastroenterology	7 (41.2)	20 (62.5)	12 (21.4)	
Cardiovascular	8 (47.1)	9 (28.1)	21 (37.5)	
Respiratory	0	3 (9.4)	0	
Infectious diseases	2 (11.8)	0	4 (7.1)	
Hematology	0	0	1 (1.8)	
Medical oncology	0	0	4 (7.1)	
Nephrology	0	0	14 (25.0)	
Length of stay in days, median (IQR)	13 (8–28)	12 (7–24)	16 (11–30)	.073
Length of stay in days (from admission till first day of assessment), median (IQR)	4 (2–6)	5 (1–7)	6 (3–17)	.026
Number of fellow patients in room, *N* (%)				
None	17 (100)		56 (100)	
1	na	7 (21.9)	na	
2	na	13 (40.6)	na	
3	na	12 (37.5)	na	
Use of night medication, *N* (%)				.890
Sleep medication	5 (29.4)	9 (28.1)	18 (32.1)	
Opioids	2 (11.8)	2 (6.3)	—	
Antipsychotics	—	—	2 (3.6)	
Chronotype, *N* (%)				.860
Early chronotype	14 (82.4)	25 (86.2)	49 (87.5)	
Normal chronotype	2 (11.8)	3 (10.3)	5 (8.9)	
Late chronotype	1 (5.9)	1 (3.4)	2 (3.6)	
EuroQol-5D-3L index, median (IQR),	0.75 (0.39–0.81)	0.81 (0.69–0.95)	0.67 (0.34–0.80)	.030
Mobility, *N* (%)				.299
I have no problems in walking about	8 (47.1)	15 (53.6)	20 (35.7)	
I have some problems in walking about	8 (47.1)	12 (42.9)	33 (58.9)	
I am confined to bed	1 (5.9)	1 (3.6)	3 (5.4)	
Self-care, *N* (%)				.149
I have no problems with self-care	11 (64.7)	21 (75.0)	30 (53.6)	
I have some problems in washing or dressing myself	6 (35.3)	6 (21.4)	22 (39.3)	
I am unable to wash or dress myself	0	1 (3.6)	4 (7.1)	
Usual activities, *N* (%)				.007
I have no problems with performing my usual activities	7 (41.2)	14 (50.0)	16 (28.6)	
I have some problems with performing my usual activities	10 (58.8)	12 (42.9)	20 (35.7)	
I am unable to perform my usual activities	0	2 (7.1)	20 (35.7)	
Pain/discomfort, *N* (%)				.255
I have no pain or discomfort	5 (29.4)	14 (50.0)	27 (48.2)	
I have moderate pain or discomfort	8 (47.1)	12 (42.9)	20 (35.7)	
I have extreme pain or discomfort	4 (23.5)	2 (7.1)	9 (16.1)	
Anxiety/depression, *N* (%)				.374
I am not anxious or depressed	14 (82.4)	18 (64.3)	35 (62.5)	
I am moderately anxious or depressed	2 (11.8)	10 (35.7)	19 (33.9)	
I am extremely anxious or depressed	1 (5.9)	0	2 (3.6)	
EuroQol-VAS, median (IQR)	50 (20–60)	51 (48–60)	60 (40–70)	.378

*Note.* IQR = interquartile range; na = not applicable; Group 1 = former hospital, without fellow patients; Group 2 = former hospital, with one to three fellow patients; Group 3 = new hospital without fellow patients.

#### RCSQ, Sleep Diary, and Accelerometry

An overview of the data from the RCSQ, sleep diary, and accelerometer is presented in Table 2. Median sleep quality according to the RCSQ total score was 70.9 (IQR 40.9–80.1) over the nights in Group 1 compared to 57.2 (IQR 36.0–76.0) for Group 2 and 65.5 (IQR 48.4–80.4) for Group 3 (*p* = .053). The median number of awakenings was similar for patients sleeping in the former hospital, respectively, 3 (IQR 2–4) for Groups 1 and 3 (IQR 1–4) for Group 2, but 2 (IQR 1–4) for Group 3 (*p* = .141). Median sleep onset latency did not differ between the two groups with SPRs (both 10 min) but was longer for Group 2 (23 min; *p* = .002). Also, sleep efficiency according to the sleep diary significantly differed between the three groups (*p* = .019). However, sleep efficiency recorded with the accelerometer did not significantly differ between the three groups (*p* = .162).

**Table 2.** Richard–Campbell Sleep Questionnaire (RCSQ) and Sleep Parameters From Sleep Diary and Accelerometer Broken Down by Room Type.

Sleep Parameters	Former Hospital Without Fellow Patients (*N* = 39 Nights)	Former Hospital With Fellow Patients (*N* = 84 Nights)	New Hospital (*N* = 157 Nights)	*p* Value^a^
RCSQ subscales, median (interquartile range [IQR])				
Depth of sleep^a^	50 (20–80)	50 (30–75)	60 (40–80)	.135
Sleep latency^a^	80 (65–84)	60 (20–81)	78 (45–90)	**.008**
Awakenings^a^	40 (30–80)	50 (30–80)	70 (48–80)	**.007**
Returning to sleep^a^	80 (60–90)	70 (30–90)	75 (40–90)	.369
Sleep quality^a^	70 (40–83)	60 (40–80)	70 (45–80)	.381
RCSQ total score				
Overall perception of sleep quality^a^	70.9 (40.9–80.1)	57.2 (36.0–76.0)	65.5 (48.4–80.4)	.053
Sleep parameters from sleep diary				
Lights out time, median (IQR)	23:00 (22:00–23:07)	23:00 (22:15–23:15)	22:45 (22:00–23:30)	.741
Sleep latency, median (IQR)	10 (5–30)	23 (10–60)	10 (5–30)	**.002**
Final wake time, median (IQR)	6:00 (5:30–7:00)	6:10 (6:00–6:45)	6:30 (6:00–7:30)	**.024**
Total sleep time (hours)^a^	7.2 (5.6–8.0)	6.5 (5.8–7.6)	6.8 (5.9–8.0)	.314
Sleep efficiency (%)^a^	83 (66–91)	78 (67–85)	85 (70–93)	**.019**
Number of awakenings^a^	3 (2–4)	3 (1–4)	2 (1–4)	.141
Sleep parameters acccelerometry				
Total sleep time (hours)^a^	8.2 (7.1–9.0)	7.7 (6.6–8.9)	8.0 (6.6–9.2)	.341
Sleep efficiency (%)^a^	70 (67–78)	67 (60–76)	67 (59–75)	.162

^a^ median [IQR].

#### Linear mixed modeling of the RCSQ and sleep efficiency

Linear mixed Model 1 with Group 3 as reference group revealed that the RCSQ total score was lowest in Group 2 but not significantly different from the reference group (*B* = −6.44, 95% CI [−13.92, 1.04], *p* = .091; Table 3). In Model 2 (with additional covariates), the RCSQ total score between the three groups did not differ significantly. Patients receiving “night medication” had significantly higher RCSQ total scores adjusting for all other covariates in the model (*p* = .040; see Table 3). In Model 1 concerning the RCSQ subscales, only scores on “sleep latency” (*p* = .022) and “awakenings” (*p* = .026) were significantly higher in Group 3 compared to Group 2 (Supplemental Tables 2–6).

We also performed linear mixed Model 1 and linear mixed Model 2 with sleep efficiency derived from accelerometer as outcome (Table 4). Sleep efficiency was not significantly different between the three groups in both models.

**Table 3.** Linear Mixed Model Analysis With Richard–Campbell Sleep Questionnaire Total Score as Outcome.

	Model 1			Model 2		
Parameter	*B*	95% CI	*p* Value	*B*	95% CI	*p* Value
Intercept	62.88	[57.10, 68.65]		65.15	[44.06, 86.23]	
Group type						
Group 1	−1.92	[−11.71, 7.86]	.698	−3.20	[−13.61, 7.21]	.544
Group 2	−6.44	[−13.92, 1.04]	.091	−6.01	[−14.27, 2.25]	.152
Group 3	Reference^a^			Reference^a^		
Night						
Night 1	−0.68	[−6.58, 5.21]	.820	0.85	[−5.11, 6.80]	.779
Night 2	0.37	[−5.63, 6.37]	.904	0.88	[−5.14, 6.90]	.774
Night 3	Reference^a^			Reference^a^		
Chronotype						
Early type				3.55	[−14.17, 21.28]	.691
Normal type				−2.04	[−23.37, 19.29]	.850
Late type				Reference^a^		
Pain (according NRS)				−0.63	[−1.86, 0.60]	.316
EQ5D index				3.38	[−10.78, 17.55]	.637
Night medication						
Yes				7.70	[0.36, 15.04]	.040
No				reference^a^		
Length of stay at the time of assessment (days) ^b^				0.07	[−0.21, 0.35]	.629

*Note.* Group 1 = former hospital, without fellow patients; Group 2 = former hospital, with one to three fellow patients; Group 3 = new hospital without fellow patients.

^a^ Reference category. ^b^ Length of stay: from admission till first day of assessment.

**Table 4.** Linear Mixed Model Analysis With Sleep Efficiency From Accelerometry as Outcome.

	Model 1			Model 2		
Parameter	*B*	95% CI	*p* Value	*B*	95% CI	*p* Value
Intercept	65.99	[62.50, 69.47]		63.15	[49.98, 76.32]	
Group type						
Group 1	3.28	[−3.07, 9.63]	0.309	2.45	[−4.38, 9.28]	0.479
Group 2	0.16	[−4.83, 5.14]	0.950	0.38	[−5.05, 5.82]	0.889
Group 3	Reference^a^			Reference^a^		
Night						
Night 1	0.10	[−2.57, 2.76]	0.944	0.23	[−2.54, 3.01]	0.870
Night 2	1.08	[−1.59, 3.75]	0.425	0.95	[−1.82, 3.73]	0.499
Night 3	Reference^a^			Reference^a^		
Chronotype						
Early type				1.95	[−9.57, 13.48]	0.736
Normal type				1.92	[−12.00, 15.85]	0.784
Late type				Reference^a^		
Pain (according NRS)				0.29	[−0.41, 1.00]	0.409
EQ5D index				−0.61	[−10.07, 8.85]	0.898
Night medication						
Yes				0.68	[−3.64, 5.00]	0.758
No				Reference^a^		
Length of stay at the time of assessment (days) ^b^				0.10	[−0.09, 0.29]	0.302

*Note.* Group 1 = former hospital, without fellow patients; Group 2= former hospital, with one to three fellow patients; Group 3 = new hospital without fellow patients.

^a^ Reference category. ^b^ Length of stay: from admission till first day of assessment.

#### Sleep disturbing factors

**Figure 2.**
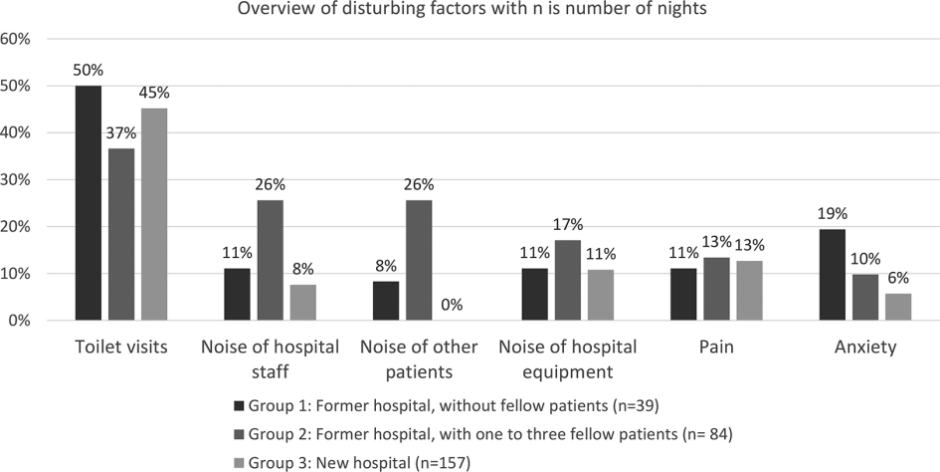
Overview of six most prevalent disturbing factors.

The prevalence of the six most prevalent reported sleep disturbing factors from the diaries is presented in Figure 2 in percentages broken down by hospital and room type. Toilet visits were the primary disturbing factor in all groups, varying from 35% to 50% of patients. The prevalence of noise nuisance from hospital staff was 8% in the new hospital compared to 26% in the former hospital with fellow patients. The prevalence of noise nuisance from other patients was 8% for patients sleeping alone in the former hospital, 26% for patients sleeping with fellow patients, and 0% in the new hospital. In more than half of the mornings, 56% and 57% of the nights in the former and new hospital, respectively, patients were woken up between 6:00 a.m. and 7:00 a.m. to check vital signs.

## Discussion

In this study, the positive impact of the new hospital environment incorporating evidence-based design features on sleep quality among hospitalized patients was lower than expected when compared to the sleep quality of patients admitted to the former hospital with multioccupancy rooms. Nevertheless, in the new hospital, patients reported significantly fewer awakenings and a significantly shorter sleep latency.

In the multivariable model with “RCSQ total score” as outcome, only the covariate “use of night medication” significantly contributed to the model, while the covariate “chronotype” did not significantly contribute to the model. This might be explained by the fact that the majority of patients in the former and new hospital were denoted as early chronotype. As chronotype has been related to age (Fischer et al., 2017), a possible explanation for this finding is the relatively older age of participants.

Also, we added self-reported pain (NRS) in the multivariable model as a covariate, but this was not related to sleep quality. This is in contrast to earlier research that do report that pain affects sleep quality (Miller et al., 2015). People with chronic pain often experience less deep sleep and they also wake up more often during the night (Whibley et al., 2019). Some patients reported use of pain medication as “night medication” in their sleep diary, but unfortunately, we did not register which pain medication was used by all participants in our study. The use of opioid-based pain medication may have affected sleep quality, since we know that sleepiness and drowsiness are common side effects of such medication (Angarita et al., 2016).

In the literature, the most reported sleep disturbing factor in hospitalized patients is noise nuisance (Delaney et al., 2019; Hedges et al., 2018; Park et al., 2014; Pulak & Jensen, 2016; Wesselius et al., 2018). As described in a 2018 systematic review, a moderate level of evidence supports that SPRs help to reduce noise nuisance and benefit perceived sleep quality (Taylor et al., 2018). We also found that in the new hospital, patients reported no noise nuisance from other patients and less noise from hospital staff compared to the former hospital. Pyrke et al. (2017) also found a significant influence from noise reducing features on sleep quality after moving to a new hospital, built according to healing environment design principles.

Besides modifiable factors such as noise nuisance, light exposure, and around-the-clock interventions, other contributors, such as illness, discomfort, and organizational factors, have been reported in the literature (Gabor et al., 2003; Le et al., 2012). Some factors may also be experienced at home; for instance, pain, nausea, anxiety, and more practically, waking up for toilet visits. Apart from illness-related and organizational factors, a different bed and an uncomfortable pillow may also be reasons for reduced sleep quality in hospitals (Gordon & Grimmer-Somers, 2011). Improvements in sleep quality in hospitalized patients could probably be achieved through interventions at an individual patient level by caregiving nurses and hospital-wide efforts to make the patients’ environment conducive to sleep (Reid, 2001). This requires optimal usage of the features that the new hospital brings, such as closed doors, lights down during the night, and optimal room temperature. The implementation of sleep hygiene bundles composed of sleep hygiene practices such as earplugs, eye mask, and a lavender scent pad had a moderate effect on sleep quality in hospitalized patients (Herscher et al., 2021). In our setting, we could also improve sleep quality in this way but also by preventing daytime napping and avoiding early morning vital signs measurements (Bellon et al., 2021; LaReau et al., 2008).

In our study, we found discrepancies between objective and subjective measured sleep duration and sleep efficiency. Sleep quality is a complex construct, and the discrepancy between subjective and objective measures of sleep may be explained by the fact that self-reported sleep and accelerometry measure different dimensions of sleep quality. In our study, sleep duration recorded with an accelerometer may be overestimated by the presumption that inactivity is interpreted as sleep. Also, it is previously reported that for older adults, perceived sleep quality is different from objective sleep quality (Landry et al., 2015). Thus, in the assessment of sleep quality, patients’ experience of sleep may be more important than some of the objective sleep parameters, such as total sleep time. Along with previous recommendations, future research should employ both subjective and objective measures to provide a more complete picture of sleep quality (Madsen et al., 2015; Moore et al., 2015; Sadeh, 2011).

### Strengths and Limitations

Several limitations of this study need to be addressed. First, the uncontrolled before-after design limits the strengths of the conclusions because other factors than the environment could have influenced the study results. Second, we did not ask about the subjects’ sleep quality at home. This means that we did not know in how far participants’ sleep quality in hospital deviated from that at home. Third, this study focused on night-time sleep only and our recommendation for future studies would be to take daytime activities and naps into account. Fourth, we did not register sleep medication adequately and some patients mentioned pain medication as sleep medication. Fifth, because the condition with multioccupancy rooms within the context of a healing environment was not available, the effects of number of patients in the room and the effects of a healing environment are difficult to distinguish from each other. Finally, although noise was mentioned as a disturbing factor, we did not objectively measure noise levels, so we do not know whether they were within an acceptable range.

## Conclusion

The present study showed that sleep quality of hospitalized patients is still a disturbing problem. Nevertheless, we found that the 100% SPRs improved sleep quality in the new hospital to a certain extent, especially compared to patients sleeping with fellow patients in the former hospital. The predominant sleep disturbing factor “noise nuisance” was reduced in the new hospital, in line with evidence-based design interventions aiming at creating a healing environment. However, the built environment is just one factor influencing sleep quality, and there are more opportunities to improve sleep quality factors such as sleep hygiene. More awareness should be created among nurses about the importance of adequate sleep and sleep hygiene. Also implementing nursing interventions such as earplugs and preventing daytime napping, and hospital-wide efforts to adjust workprocesses and common practices to minimize sleep disturbing may improve sleep quality.

***the built environment is just one factor influencing sleep quality, and there are more opportunities to improve sleep quality factors such as sleep hygiene***

## Implications for Practice

- Sleep is a multifaceted construct and future research should employ both subjective and objective measures to provide a more complete picture of sleep quality.
- SPRs may improve sleep quality because of noise level control but have no effect on illness-related factors such as pain and nausea.
- Nighttime measures and early awakenings should be considered carefully in promoting sleep quality.

## Supplemental Material

Supplemental Material, sj-pdf-1-her-10.1177_19375867231168895 - Hospitalized Patients’ Sleep Quality Compared Between Multioccupancy Rooms and Single-Patient Rooms Click here for additional data file. Supplemental Material, sj-pdf-1-her-10.1177_19375867231168895 for Hospitalized Patients’ Sleep Quality Compared Between Multioccupancy Rooms and Single-Patient Rooms by Laura Schafthuizen, Erwin Ista, Marianne van der Heijden, Liesbeth van Heel, Jill Maben, Joost van Rosmalen, Casper H. J. van Eijck and Monique van Dijk in HERD: Health Environments Research & Design Journal

Supplemental Material, sj-pdf-2-her-10.1177_19375867231168895 - Hospitalized Patients’ Sleep Quality Compared Between Multioccupancy Rooms and Single-Patient Rooms Click here for additional data file. Supplemental Material, sj-pdf-2-her-10.1177_19375867231168895 for Hospitalized Patients’ Sleep Quality Compared Between Multioccupancy Rooms and Single-Patient Rooms by Laura Schafthuizen, Erwin Ista, Marianne van der Heijden, Liesbeth van Heel, Jill Maben, Joost van Rosmalen, Casper H. J. van Eijck and Monique van Dijk in HERD: Health Environments Research & Design Journal
