# Supplementary material for: Hospitalized Patients’ Sleep Quality Compared Between Multioccupancy Rooms and Single-Patient Rooms
Source: HERD. 2023 May 4;16(3):119–33. doi: 10.1177/19375867231168895 (PMC10328146; doi:10.1177/19375867231168895)
Supplement: Supplemental Material, sj-pdf-1-her-10.1177_19375867231168895 - Hospitalized Patients’ Sleep Quality Compared Between Multioccupancy Rooms and Single-Patient Rooms [file sj-pdf-1-her-10.1177_19375867231168895.pdf]

Supplemental table 1. Sleep parameters measured with the RCSQ, sleep diary, and accelerometer

	**Sleep latency**	**Awakenings**	**Sleep depth**	**Returning to sleep**	**Sleep quality**	**Sleep quality 'total score'**	**Sleep efficiency**	**Sleep duration**
**Richards-Campbell Sleep Questionnaire**	Last night, the first time I got to sleep, I: Score 0=just never could fall asleep… Score 100=fell asleep almost immediately.	Last night, I was: Score 0=awake all night long …Score 100=awake very little.	My sleep last night was: Score 0=light sleep…Score 100=deep sleep.	Last night, when I woke up or was awakened, I: Score 0=could not get back to sleep…Score 100=got back to sleep immediately.	I would describe my sleep last night as: Score 0=a bad night's sleep …Score 100=a good night's sleep.	The 'total score', represents the overall perception of sleep quality.	NA	NA
**Sleep diary**	How long did it take to fall asleep? (minutes)	How many times did you wake up last night?	NA	NA	NA	NA	The ratio of total sleep time to time in bed (multiplied by 100 to yield a percentage)	Time asleep in hours between 'lights out' and waking up time, minus sleep latency
**Accelerometer**	NA	NA	NA	NA	NA	NA	Sleep episodes were defined as the sustained periods of inactivity. From this, sleep efficiency was calculated as the percentage of time asleep within the Sleep Period Time (SPT)-window.	The sum of all nocturnal sleep bouts.
