# Supplementary material for: Hospitalized Patients’ Sleep Quality Compared Between Multioccupancy Rooms and Single-Patient Rooms
Source: HERD. 2023 May 4;16(3):119–33. doi: 10.1177/19375867231168895 (PMC10328146; doi:10.1177/19375867231168895)
Supplement: Supplemental Material, sj-pdf-2-her-10.1177_19375867231168895 - Hospitalized Patients’ Sleep Quality Compared Between Multioccupancy Rooms and Single-Patient Rooms [file sj-pdf-2-her-10.1177_19375867231168895.pdf]

SUPPLEMENTAL TABLES 2 – 6

Table 2. Linear mixed model analysis with sleep depth as outcome

	Model 1		
Parameter	B	95% CI	p-value
Intercept	61.18	54.32 to 68.04	
Group type			
Group 1	-7.33	-18.75 to 4.09	0.206
Group 2	-5.94	-14.58 to 2.70	0.176
Group 3	reference [a]		
Night			
Night 1	-4.10	-11.46 to 3.25	0.272
Night 2	-2.26	-9.74 to 5.22	0.551
Night 3	reference [a]		

[a] Reference category.

Table 3. Linear mixed model analysis with sleep latency as outcome

	Model 1		
Parameter	B	95% CI	p-value
Intercept	65.50	58.13 to 72.87	
Group type			
Group 1	3.36	-9.07 to 15.80	0.594
Group 2	-11.09	-20.55 to -1.63	**0.022**
Group 3	reference [a]		
Night			
Night 1	-0.25	-7.90 to 7.40	0.949
Night 2	0.47	-7.30 to 8.23	0.906
Night 3	reference [a]		

[a] Reference category.

Table 4. Linear mixed model analysis with awakenings as outcome

	Model 1		
Parameter	B	95% CI	p-value
Intercept	65.86	56.04 to 69.69	
Group type			
Group 1	-9.95	-21.56 to 1.66	0.092
Group 2	-10.10	-19.0 to -1.20	**0.026**
Group 3	reference [a]		
Night			
Night 1	-0.38	-7.26 to 6.49	0.913

Night 2	-0.04	-7.05 to 6.96	0.990
Night 3	reference [a]		

Table 5. Linear mixed model analysis with returning to sleep as outcome

	Model 1		
Parameter	B	95% CI	p-value
Intercept	65.21	57.58 to 72.84	
Group type			
Group 1	4.01	-9.06 to 17.09	0.545
Group 2	-3.42	-13.46 to 6.62	0.501
Group 3	reference [a]		
Night			
Night 1	1.41	-6.09 to 8.91	0.711
Night 2	-0.09	-7.70 to 7.51	0.980
Night 3	reference [a]		

[a] Reference category.

Table 6. Linear mixed model analysis with sleep quality as outcome

	Model 1		
Parameter	B	95% CI	p-value
Intercept	59.82	53.21 to 66.42	
Group type			
Group 1	0.42	-10.86 to 11.70	0.942
Group 2	-2.06	-10.71 to 6.58	0.637
Group 3	reference [a]		
Night			
Night 1	-0.14	-6.69 to 6.41	0.966
Night 2	3.39	-3.26 to 10.04	0.316
Night 3	reference [a]		

[a] Reference category.
